# Assessment of the quality and content of clinical practice guidelines (CPGs) for vitamin D and for immigrants using the AGREE-II instrument: a protocol for systematic review

**DOI:** 10.1186/s13643-022-02129-6

**Published:** 2022-11-17

**Authors:** Said Yousef, Lamia Hayawi, Douglas Manuel, Ian Colman, Manny Papadimitropoulos, Alomgir Hossain, Moez AlIslam Faris, George A. Wells

**Affiliations:** 1 School of Epidemiology and Public Health, Faculty of Medicine, University of Ottawa, Ottawa, Canada; 2 Cardiovascular Research Methods Centre, University of Ottawa Heart Institute, Room H1281, 40 Ruskin Street, Ottawa, ON K1Y4W7 Canada; 3 Children’s Hospital of Eastern Ontario Research Institute, Ottawa, ON Canada; 4 Ottawa Hospital Research Institute, Ottawa, Canada; 5 Institute for Clinical Evaluative Sciences, Ottawa, Canada; 6 Department of Family Medicine, University of Ottawa, Ottawa, Canada; 7 Eli Lilly Canada Inc., Toronto, Canada; 8 Faculty of Pharmacy, University of Toronto, Toronto, Canada; 9 Department of Clinical Nutrition and Dietetics, College of Health Sciences/Research Institute of Medical and Health Sciences (RIMHS), University of Sharjah, Sharjah, UAE

**Keywords:** Vitamin D, Clinical practice guideline, Immigrants, AGREE-II

## Abstract

**Introduction:**

Worldwide, more immigrants experience vitamin D (vitD) deficiency than non-immigrants, which is attributed to ethnic variations, place or region of birth, skin pigmentation, clothing style, and resettlement-related changes in diet, physical activity, and sun exposure. Current recommendations in clinical practice guidelines (CPGs) concerning vitD are inadequate to address vitD deficiency among immigrants. CPGs may also lack guidance for physicians on vitD supplementation for immigrants. Moreover, there are concerns about the overall quality of these CPGs.

**Objectives:**

This systematic review will collate and critically appraise CPGs relevant to immigrants’ health and vitD. Moreover, we will evaluate whether the CPGs of vitD including recommendations for immigrants and clarify whether the CPGs of immigrants include recommendations on vitD.

**Methods:**

A systematic search of Ovid MEDLINE® ALL, EMBASE, and Turning Research Into Practice (TRIP) electronic databases, guideline repositories, and gray literature will be conducted to identify relevant CPGs. Two reviewers will independently evaluate the methodological quality of the retrieved guidelines using the Appraisal of Guidelines, Research, and Evaluation-II (AGREE-II) instrument. CPGs scoring ≥60% in at least four domains, including “rigor of development,” will be considered high quality.

**Conclusion:**

Evaluating the quality and content of relevant CPGs may support researchers in developing national and global guidelines for immigrants. Furthermore, it may support vitD testing, nutritional counseling, and supplementation for vulnerable immigrant sub-populations.

**Systematic review registration:**

PROSPERO CRD42021240562.

**Supplementary Information:**

The online version contains supplementary material available at 10.1186/s13643-022-02129-6.

## Introduction

Globally, immigrant populations represent diverse categories including economic migrants, family members, refugees, and asylum [1, 2]. Immigrants’ health status after immigration remains an important area of concern [3, 4]. Studies have shown that immigrants’ physical and mental health declines quickly after arrival to their host countries [1, 4–6], and further health changes can occur over 5–10 years [4, 7]. Recent migrants are reported to have better health compared with people of the host country, including long-term migrants who had migrated to the host country at an earlier time; this phenomenon is called the “healthy immigrant effect” [8]. It has been hypothesized that the healthy immigrant effect results from a combination of pre- and post-migration factors. Pre-migration factors include biased and rigorous selection criteria that favor immigrants that are healthier, wealthier, and better educated than the general population in the country of origin [8, 9]. In addition, a decline in immigrants’ health status after arrival may be explained by psychosocial factors and lifestyle changes [9–11]. The mechanisms underlying this health decline in immigrants have not been explicitly identified [4]. However, the healthy immigrant effect appears to decline over time when immigrants are exposed to their new environment [5]. Evidence also suggests that immigrants have lower vitD compared with native-born populations [12–14]. In a recent national large-scale Canadian data, ethnicity was found the most strongly associated factor with serum vitD status among immigrants from 153 countries and more than 13 ethnicities [14]. Moreover, vitD status varies among immigrants because of skin pigmentation, clothing, place or region of birth, and resettlement changes in diet, physical activity, and sun exposure [12, 14–16].

The Institute of Medicine (IOM) defines clinical practice guidelines (CPGs) as “statements that include recommendations intended to optimize patient care that are informed by a systematic review of evidence and an assessment of the benefits and harms of alternative care options” [17]. The main purposes of CPGs are to improve the effectiveness and quality of care and decrease variations in healthcare practices based on scientific evidence [17, 18]. Worldwide, more CPGs covering immigrants’ health have become available in the last two decades, with the main focus areas being screening, treatment, immunization, infectious diseases, mental health, and chronic diseases [2, 19, 20]. The Canadian Immigrant and Refugee Guideline refers to the first 5 years of immigration to Canada as the time during which the healthy immigrant effect begins to decline. However, the developers of this guideline highlighted the topic of vitD deficiency among immigrants as an emerging condition that was not emphasized in their guidelines [20].

CPGs covering vitD that are currently available vary among countries and professional development organizations in terms of the scope, targeted population, and recommendations [21–24]. Notably, the current recommendations for vitD are inadequate to address the growing epidemic of vitD deficiency in immigrant populations [23, 25]. Moreover, the majority of guidelines do not include specific recommendations for different immigrant populations or certain ethnic groups [21, 23, 26, 27].

In addition, concerns have been raised about the overall quality of these CPGs [2, 28, 29]. The Appraisal of Guidelines for Research and Evaluation-II (AGREE-II) instrument has been widely used to assess guideline quality, define the information that needs to be reported, and inform guideline development [30, 31]. The overall guideline assessment includes judgment on the quality of the guideline and whether the guideline would be recommended for use [31]. Previous studies that assessed the quality of CPGs using the AGREE-II instrument were inconsistent in how they determined high quality. For example, with the inclusion of the “rigor of development” domain, a score of high quality was reported as ≥60% in at least three of the six domains [2], four of the six domains [32], or in all six domains [33].

### Research objectives

This systematic review will focus on the critical appraisal of guidelines relevant to vitD and immigrants’ health using the AGREE-II instrument. Moreover, it aims to evaluate the guideline content to clarify whether recommendations for immigrant populations were included in vitD guidelines and whether vitD recommendations were included in immigrant guidelines.

### Research questions

The appraisal will be based on specific research questions and clarify whether the included CPGs comply with the quality standards of the AGREE-II instrument. The research questions are as follows: “Do immigrant CPGs include recommendations on vitD?” and “Do vitD CPGs include recommendations for immigrants, including refugees and asylum seekers?”

## Methods and study design

The protocol for this review was registered with PROSPERO (CRD42021240562) [34]. The review procedures will follow the methods outlined in the Cochrane Handbook for Systematic Reviews [35]. This protocol followed the Preferred Reporting Items for Systematic Reviews and Meta-Analyses Protocol (PRISMA-P) Statement [36]; the checklist is presented in Additional file 1.

### Search strategy

The search strategy used to identify published CPGs covering immigrants and vitD will be developed in consultation with an experienced medical information specialist. The databases searched will include Ovid MEDLINE® ALL and EMBASE. These searches will use a combination of controlled vocabulary (e.g., “VitD,” “emigrants and immigrants,” “refugees”) and keywords (e.g., “vitD,” “asylum seeker,” “foreigner”) and incorporate a CADTH-derived guideline filter. The Ovid MEDLINE search strategy is presented in Additional file 2.

We will also search the Turning Research Into Practice (TRIP) database. In addition, we will search gray literature for guidelines that were internally produced by governments, organizations, and academic institutions and that publishing is a secondary activity for these organizations. For example, a search in the Guidelines International Network (GIN), Joanna Briggs Institute (JBI), World Health Organization (WHO), Healthcare Research and Quality (AHRQ), and National Institution for Excellence (NICE) will be conducted. Results will be limited to relevant publications between 2010 and the date of the search. Supplemental searching will be performed on the reference lists of identified CPGs to detect other key articles.

### Eligibility criteria

We will include two types of CPGs: those concerning immigrants’ health and those concerning vitD. The included guidelines will conform to the IOM definition of CPGs [17] and must have been published in the English language between 2010 and the search date. We will include CPGs that have recommendations intended for healthcare professionals on screening, diagnosis, management, or treatment related to immigrants’ health or vitD. No restrictions will be applied in regard to age, sex, or health status. In this review, we will define immigrants as those who move cross-country or are international migrants residing in a destination country, irrespective of legal status and generation. Therefore, any CPGs that include immigrants as a primary population will be evaluated using the AGREE-II tool. CPGs for vitD that emphasizes care for patients who are at risk for vitD deficiency in terms of evaluation, treatment, and prevention will also be evaluated using the AGREE-II tool.

### Exclusion criteria

We will exclude non-English language guidelines or incomplete texts. We will also exclude healthcare guidelines that do not consider immigrants or vitD as the primary objective. Dietary or nutritional guidelines for vitD that are not intended for healthcare practices or that target the general population will also be excluded. Furthermore, we will exclude position and consensus papers as well as any other documents that are not equivalent to guidelines.

### Outcomes

The primary outcome of this review will be the quality score of the included CPGs based on the AGREE-II scoring tool. Secondary outcomes will be recommendations on vitD as part of immigrant guidelines and recommendations in vitD guidelines that target immigrants.

### Selection of CPGs and data extraction

The systematic review software “Covidence” (www.covidence.org) will be used to remove duplicates and screen the titles and abstracts of identified GPGs. We will use the AGREE-II instrument (https://www.agreetrust.org) to evaluate the quality of the included guidelines. This tool comprises 23 items and evaluates six domains: “scope and purpose,” “stakeholder involvement,” “rigor of development,” “clarity of presentation,” “applicability,” and “editorial independence” [31]. An electronic standardized form will be used to record these data and individual items will be rated using a seven-point Likert scale (1 = strongly disagree, 7 = strongly agree) [31]. More details are available in the AGREE-II manual [31]. The AGREE-II scoring sheet is presented in Additional file 3.

Two reviewers (SY and LH) will independently follow the inclusion and exclusion criteria to assess the citations for inclusion. Both reviewers will resolve differences in their decisions through discussion. A consultation with a third reviewer will be counted when a consensus cannot be reached.

One reviewer (SY) will extract the main characteristics of the included published guidelines, including the title, author(s) name(s), year of publication, country, and organization that produced the guideline, and key recommendations related to immigrants in vitD CPGs or vitD in immigrant CPGs. The extracted data will be checked by a second reviewer.

Following the application of the inclusion and exclusion criteria, two reviewers (SY and LH) will independently evaluate the methodological quality of the included guidelines. Both reviewers had previous training in using the online AGREE-II, and the resulting study was published [32]. Any disagreements will be resolved by discussion with a third reviewer to reach a consensus. The reviewers will evaluate the content of each CPG, and any additional supporting documents that are cited in the published guidelines.

### Quality evaluation and data synthesis

The reviewers’ decisions on the overall quality of recommendations contained in the guidelines will be based on the context in which the AGREE-II is being used. The mean scores for each AGREE-II domain and the overall quality will be presented. A standardized score for each domain will be calculated using the formula: ((actual score—minimum score)/(maximum score—minimum score)) × 100% [37].

CPGs that score ≥60% in at least four of the six domains, including the “rigor of development” domain, will be considered high quality [32, 38–40]. The intra-class correlation coefficient (ICC) and 95% confidence intervals will be calculated to assess the overall agreement between the reviewers for each guideline. The ICC will be classified as poor (<0.40), moderate (0.40–0.59), good (0.60–0.74), or excellent (0.75–1.00) based on a previous study [41]. We will analyze and summarize the extracted data in a tabular and narrative format. The characteristics of the identified CPGs including author, year, and country with the title will be presented in a table and quality assessment information like the domain percentage score, the ICC, and the overall quality of each guideline in another table. We will narratively discuss if immigrants or ethnicity was mentioned in the guidelines of VitD. Similarly, we will narratively discuss if vitD D was mentioned in CPGs for immigrants.

## Discussion

Current national immigrant health policies only cover fragmented snapshots of global immigrant health. In response to growing issues related to immigrants’ health, previous researchers recommended regional or global health-related protection agreements [42]. Furthermore, researchers recommended developing a global immigrant guideline that specifies sub-populations for which the evidence and recommendations may differ from the overall immigrant population [12, 43]. For example, the Endocrine Society guidelines recommend screening African Americans, Hispanic Americans [44], and all refugees for vitD deficiency [45]. In a recent Canadian national survey analysis, Yousef et al. (2021) reported vitD levels among immigrants from 153 origins and more than 13 ethnic groups. That study reported vitD deficiency attributable to ethnicity or country of birth was 6.6–62.1%, with the highest deficiency levels in the Japanese, Arab, and Southeast Asian ethnic groups, and among those born in Morocco, India, and Lebanon [14].

In this review, evaluating the quality and reviewing the content of the guidelines in terms of the recommendations for vitD and immigrants may support researchers in developing national and global immigrant guidelines. VitD-related recommendations may differ for sub-populations who are at high risk for vitD deficiency. Moreover, this review may also help and guide clinicians to support specific vitD testing, nutritional counseling, and supplementation for vulnerable immigrant sub-groups.

## Supplementary Information

**Additional file 1.** PRISMA-P checklist-Vit D Yousef et al..

**Additional file 2.** Ovid Medline CPGs.

**Additional file 3.** AGREE-II Checklist.

## Data Availability

Not applicable.

## Abbreviations

AGREE-II: Appraisal of Guidelines, Research, and Evaluation II instrument
CPGs: Clinical practice guidelines
VitD: Vitamin D
IOM: Institute of Medicine
PRISMA-P: Preferred Reporting Items for Systematic Reviews and Meta-Analyses Protocol
CADTH: Canadian Agency For Drugs And Technologies In Health
TRIP: Turning Research Into Practice
ICC: Intra-class correlation coefficient
