# Supplementary material for: Assessment of the quality and content of clinical practice guidelines (CPGs) for vitamin D and for immigrants using the AGREE-II instrument: a protocol for systematic review
Source: Syst Rev. 2022 Nov 17;11:245. doi: 10.1186/s13643-022-02129-6 (PMC9673290; doi:10.1186/s13643-022-02129-6)
Supplement: Supplementary file 2 — Additional file 2. Ovid Medline CPGs. [file 13643_2022_2129_MOESM2_ESM.pdf]

Additional file 2:

Ovid MEDLINE(R) ALL 1946 to October 14, 2020
Date Run: October 15, 2020

1	exp Vitamin D/
2	vitamin D.tw.
3	exp vitamin d deficiency/
4	((avitaminosis or hypovitaminosis) adj1 (D or D2 or D3)).tw,kf.
5	or/1-4
6	exp "Emigrants and Immigrants"/
7	"Emigration and Immigration"/
8	"Transients and Migrants"/
9	refugees/
10	asylum seeker/
11	(alien? or emigrat* or emigrant? or foreigner? or immigrat* or immigrant? or migrant? or migrate? or migration? or migrating or undocumented worker? or foreign born or refugee? or asylum seeker?).tw.
12	or/6-11
13	(guideline or practice guideline or consensus development conference or consensus development conference, NIH).pt.
14	(guideline* or standards or consensus* or recommendat*).ti.
15	(practice parameter* or position statement* or policy statement* or CPG or CPGs or best practice*).ti.
16	(care adj2 (path or paths or pathway or pathways or map or maps or plan or plans or standard)).ti.
17	((critical or clinical or practice) adj2 (path or paths or pathway or pathways or protocol*)).ti.
18	(algorithm* and (pharmacotherap* or chemotherap* or chemotreatment* or therap* or treatment* or intervention*)).ti.
19	(algorithm* and (screening or examination or test or tested or testing or assessment* or diagnosis or diagnoses or diagnosed or diagnosing)).ti.
20	or/13-19
21	(5 or 12) and 20

22	exp animals/
23	exp animal experimentation/ or exp animal experiment/
24	exp models animal/
25	nonhuman/
26	exp vertebrate/ or exp vertebrates/
27	exp humans/
28	exp human experimentation/ or exp human experiment/
29	or/22-26
30	or/27-28
31	29 not 30
32	21 not 31
33	limit 32 to yr="2010 - 2020"
34	(conference abstract or conference review).pt.
35	33 not 34
