# Supplementary material for: Assessment of the quality and content of clinical practice guidelines (CPGs) for vitamin D and for immigrants using the AGREE-II instrument: a protocol for systematic review
Source: Syst Rev. 2022 Nov 17;11:245. doi: 10.1186/s13643-022-02129-6 (PMC9673290; doi:10.1186/s13643-022-02129-6)
Supplement: Supplementary file 3 — Additional file 3. AGREE-II Checklist. [file 13643_2022_2129_MOESM3_ESM.pdf]

Additional file 2
AGREE II Score Sheet

Domain	Item	AGREE II Rating						
		1 *Strongly Disagree*	2	3	4	5	6	7 *Strongly Agree*
Scope and purpose	1. The overall objective(s) of the guideline is (are) specifically described.							
	2. The health question(s) covered by the guideline is (are) specifically described.							
	3. The population (patients, public, etc.) to whom the guideline is meant to apply is specifically described.							
Stakeholder involvement	4. The guideline development group includes individuals from all the relevant professional groups.							
	5. The views and preferences of the target population (patients, public, etc.) have been sought.							
	6. The target users of the guideline are clearly defined.							
Rigor of development	7. Systematic methods were used to search for evidence.							
	8. The criteria for selecting the evidence are clearly described.							
	9. The strengths and limitations of the body of evidence are clearly described.							
	10. The methods for formulating the recommendations are clearly described.							
	11. The health benefits, side effects and risks have been considered in formulating the recommendations.							
	12. There is an explicit link between the recommendations and the supporting evidence.							
	13. The guideline has been externally reviewed by experts prior to its publication.							
	14. A procedure for updating the guideline is provided.							
Clarity of presentation	15. The recommendations are specific and unambiguous.							
	16. The different options for management of the condition or health issue are clearly presented.							
	17. Key recommendations are easily identifiable.							
Applicability	18. The guideline describes facilitators and barriers to its application.							
	19. The guideline provides advice and/or tools on how the recommendations can be put into practice.							
	20. The potential resource implications of applying the recommendations have been considered.							
	21. The guideline presents monitoring and/ or auditing criteria.							
Editorial independence	22. The views of the funding body have not influenced the content of the guideline.							
	23. Competing interests of guideline development group members have been recorded and addressed.							
Overall Guideline Assessment	1. Rate the overall quality of this guideline.	1 *Lowest possible quality*	2	3	4	5	6	7 *Highest possible quality*
Overall Guideline Assessment	2. I would recommend this guideline for use.	*Yes*	*Yes, with modifications*					*No*
